# Aβ43 aggregates exhibit enhanced prion-like seeding activity in mice

**DOI:** 10.1186/s40478-021-01187-6

**Published:** 2021-05-10

**Authors:** Alejandro Ruiz-Riquelme, Alison Mao, Marim M. Barghash, Heather H. C. Lau, Erica Stuart, Gabor G. Kovacs, K. Peter R. Nilsson, Paul E. Fraser, Gerold Schmitt-Ulms, Joel C. Watts

**Affiliations:** 1 Tanz Centre for Research in Neurodegenerative Diseases, University of Toronto, Krembil Discovery Tower, Rm. 4KD481, 60 Leonard Ave., Toronto, ON M5T 0S8 Canada; 2 Department of Biochemistry, University of Toronto, Toronto, ON Canada; 3 Department of Laboratory Medicine and Pathobiology, University of Toronto, Toronto, ON Canada; 4 Laboratory Medicine Program and Krembil Brain Institute, University Health Network, Toronto, ON Canada; 5 Department of Physics, Chemistry, and Biology, Linköping University, Linköping, Sweden; 6 Department of Medical Biophysics, University of Toronto, Toronto, ON Canada; 7 Present Address: UK Dementia Research Institute, University College London, London, UK

**Keywords:** Alzheimer’s disease, Prion-like propagation, Amyloid-β, Knock-in mice, Strains

## Abstract

When injected into genetically modified mice, aggregates of the amyloid-β (Aβ) peptide from the brains of Alzheimer’s disease (AD) patients or transgenic AD mouse models seed cerebral Aβ deposition in a prion-like fashion. Within the brain, Aβ exists as a pool of distinct C-terminal variants with lengths ranging from 37 to 43 amino acids, yet the relative contribution of individual C-terminal Aβ variants to the seeding behavior of Aβ aggregates remains unknown. Here, we have investigated the relative seeding activities of Aβ aggregates composed exclusively of recombinant Aβ38, Aβ40, Aβ42, or Aβ43. Cerebral Aβ42 levels were not increased in *App*^NL−F^ knock-in mice injected with Aβ38 or Aβ40 aggregates and were only increased in a subset of mice injected with Aβ42 aggregates. In contrast, significant accumulation of Aβ42 was observed in the brains of all mice inoculated with Aβ43 aggregates, and the extent of Aβ42 induction was comparable to that in mice injected with brain-derived Aβ seeds. Mice inoculated with Aβ43 aggregates exhibited a distinct pattern of cerebral Aβ pathology compared to mice injected with brain-derived Aβ aggregates, suggesting that recombinant Aβ43 may polymerize into a unique strain. Our results indicate that aggregates containing longer Aβ C-terminal variants are more potent inducers of cerebral Aβ deposition and highlight the potential role of Aβ43 seeds as a crucial factor in the initial stages of Aβ pathology in AD.

**Supplementary Information:**

The online version contains supplementary material available at 10.1186/s40478-021-01187-6.

## Introduction

Alzheimer’s disease (AD) is a progressive neurodegenerative disorder of ageing and is the most common cause of dementia in humans. The brains of AD patients contain two hallmark pathologies: extracellular amyloid plaques containing aggregated amyloid-β (Aβ) peptide and intracellular neurofibrillary tangles composed of aggregated and hyperphosphorylated tau protein. Some AD cases also feature Aβ deposition within cerebral blood vessels, referred to as Aβ cerebral amyloid angiopathy (CAA). One hypothesis for the molecular sequence of events in AD, the amyloid cascade hypothesis, speculates that the polymerization and deposition of Aβ peptide is the initiating event in the disease, which stimulates the downstream aggregation and deposition of tau within neurons [80]. The Aβ peptide is produced by cleavage of the amyloid precursor protein (APP) by β- and γ-secretase, and mutations within the genes encoding APP or γ-secretase components that increase Aβ levels or augment its aggregation potential cause early-onset familial forms of AD, arguing that Aβ aggregation is central to AD pathogenesis.

In AD, both Aβ and tau exhibit hierarchical patterns of deposition within the brain [6, 88]. A prion-like mechanism in which pre-existing Aβ “seeds” template the addition of monomeric Aβ to growing Aβ aggregates has been proposed to explain the apparent spreading of Aβ aggregates within the brains of AD patients [31, 67]. In support of this theory, intracerebral or peripheral inoculation of transgenic mice expressing mutant or wild-type human APP with brain extracts rich in Aβ aggregates induces the cerebral deposition of Aβ in a prion-like fashion [19, 32, 55, 57]. Aβ aggregates purified from the brains of transgenic AD mouse models or composed exclusively of synthetic Aβ peptides are sufficient to induce Aβ pathology in recipient mice, demonstrating that Aβ aggregates themselves are responsible for the prion-like transmission of Aβ pathology [83, 84]. While Aβ pathology is transmissible in genetically modified mice, primates [71], and potentially in humans exposed to Aβ-contaminated growth hormone preparations or dura mater grafts [3, 18, 21, 23, 25, 30, 39, 68, 72], there is currently no evidence that the full clinicopathological spectrum of AD can be transmitted from person-to-person [2, 49].

The precise species of Aβ aggregates that mediates their prion-like seeding behavior remains unknown. Within the brain, Aβ aggregates can vary greatly in size, ranging from dimers to oligomers to fibrils. While there is evidence that soluble and/or oligomeric Aβ assemblies exhibit seeding activity in mice [20, 33, 43], it is clear that larger, protease-resistant fibrillar Aβ species are also effective at inducing cerebral Aβ deposition [43, 84]. Furthermore, the Aβ peptide itself is heterogeneous, with variability in the primary amino acid sequence existing at both the N- and C-terminal ends. Aβ variants with C-termini that terminate between residues 37 and 43 of the cognate Aβ sequence are generated due to differential cleavage of membrane-embedded APP-derived fragments by presenilin proteins, the catalytic components of the γ-secretase complex [4, 41, 86]. Longer Aβ peptides such as Aβ42 and Aβ43 are more aggregation-prone and are typically found within the cores of amyloid plaques [29, 76], whereas shorter peptides such as Aβ38 and Aβ40 are found deposited within the periphery of dense-core plaques and constitute the principal components of Aβ CAA [17, 58, 92].

An additional level of complexity is that Aβ aggregates can exist as conformationally distinct “strains” [37, 45, 52, 53, 63, 64, 66, 69], some of which are capable of inducing the formation of morphologically distinct Aβ deposits when injected into mice [13, 15, 24, 70, 83, 94]. Interestingly, Aβ aggregates present in brains from AD patients or transgenic AD mouse models exhibit much higher seeding activity in mice than Aβ aggregates composed of synthetic Aβ [55, 84]. One potential explanation is that synthetic and brain-derived Aβ aggregates are structurally distinct. Indeed, the structure of CAA-associated Aβ40 aggregates purified from an AD brain is markedly different from those obtained via the polymerization of synthetic Aβ in vitro [38].

We recently demonstrated that intracerebral injection of *App*^NL−F^ knock-in mice with brain-derived Aβ aggregates results in the robust induction of cerebral Aβ deposition [73]. These mice represent an ideal paradigm for assessing the prion-like seeding behavior of Aβ aggregates since they lack artifacts associated with APP over-expression, and APP is expressed with the correct spatiotemporal pattern within the brain [74, 75]. In this study, we used *App*^NL−F^ mice and recombinant Aβ species to compare the relative seeding activities of Aβ aggregates composed exclusively of individual Aβ C-terminal peptide variants. Unexpectedly, we found that recombinant Aβ43 aggregates were uniquely able to induce cerebral Aβ deposition with an efficiency comparable to brain-derived Aβ aggregates.

## Materials and methods

### Production of protease-resistant recombinant Aβ aggregates

Stocks (0.5 mg) of recombinant Aβ1-38, Aβ1-40, Aβ1-42 and Aβ1-43 peptides were purchased from rPeptide (catalog numbers A-1078-1, A-1001-1, A-1002-1, and A-1005-1, respectively). Peptides were dissolved in hexafluoroisopropanol (HFIP), separated into 50 µg aliquots, and then HFIP was evaporated overnight. Peptide aliquots were stored at −80 ºC. For production of aggregates, 50 µg of dried peptide film was resuspended in 20 µL DMSO, diluted with 480 µL 10 mM sodium phosphate (NaP) buffer (pH 7.4), and then Aβ was quantified by measuring absorbance at 280 nm using a Nanodrop ND-1000 spectrophotometer. Aβ samples in 1.5 mL tubes were diluted to 5 µM in NaP buffer and then 800 µL aliquots were incubated at 37 °C for 72 h in an Eppendorf Thermomixer with continuous shaking at 900 rpm. For samples that were used for inoculation of mice, Aβ aggregates were treated with 0.5 µM (14.5 µg/mL) proteinase K (PK) (Thermo Scientific #EO0491) for 1 h at 37 °C with shaking at 600 rpm, and then the reaction was halted by the addition of 2 mM PMSF. Samples were centrifuged at 100,000 × *g* (48,000 rpm) for 1 h at 4 ºC in a TLA-55 rotor (Beckman), and then the pellets were resuspended in dH_2_O and stored at − 30 ºC. The concentration of the PK-resistant recombinant Aβ aggregates was determined using an Aβ_1-x_ ELISA kit (IBL America #27729) following treatment with formic acid.

### Thioflavin T aggregation assays

Recombinant Aβ samples (5 µM in NaP buffer) were kept on ice and then 20 µM Thioflavin T (ThT; Sigma-Aldrich #T3516) was added to a final concentration of 20 µM. Aliquots of 100 µL were placed in black 96-well clear bottom plates (Nunc #265301) and then incubated in a microplate plate reader (BMG CLARIOstar) set at 37 ºC. Samples were subjected to rounds of 1 min rest and 4 min shaking (double orbital, 700 rpm), and ThT fluorescence (excitation: 444 ± 5 nm; emission: 485 ± 5 nm) was measured every 5 min.

### Conformational stability assays

Aβ conformational stability assays were performed essentially as previously described [46]. Briefly, aliquots of guanidine hydrochloride (GdnHCl) stocks (30 µL) were added to 10 µL of 5 µM recombinant Aβ aggregates to give final GdnHCl concentrations of 1, 2, 2.5, 3, 3.5, 4, 4.5, 5, 5.5, or 6 M. Samples were incubated at room temperature for 2 h with shaking (800 rpm) and then diluted to 0.4 M GdnHCl in PBS containing 0.5% (w/v) sodium deoxycholate and 0.5% (v/v) NP-40 to a final volume of 600 µL. Samples were then treated with 20 µg/mL PK for 1 h at 37 ºC with shaking (600 rpm). Digestions were stopped by the addition of 2 mM PMSF, and then sarkosyl was added to a final concentration of 2% (v/v). Following ultracentrifugation at 100,000 × *g* for 1 h at 4 ºC in a TLA-55 rotor, pellets were resuspended in 100 µL of formic acid, vortexed, and then sonicated in a water bath sonicator for 10 min. The formic acid was evaporated using a speed-vac for 30 min, and then dried pellets were resuspended in 1X Bolt LDS loading buffer and boiled for 10 min. Samples were then analyzed by immunoblotting as described below.

### PK digestion of recombinant Aβ aggregates

Aliquots of 5 µM recombinant Aβ aggregates (5 µL) were treated with various concentrations of PK in a final volume of 20 µL PBS containing 0.5% (w/v) sodium deoxycholate and 0.5% (v/v) NP-40. Digestions were performed for 1 h at 37 ºC with shaking (600 rpm). Digestions were stopped by the addition of 2 mM PMSF, and then PBS containing 2% (v/v) sarkosyl was added to generate a final volume of 200 µL. Samples were then ultracentrifuged and processed identically as described above for the conformational stability assays.

### Dye-binding assays

Dye-binding assays were performed essentially as described previously [44]. Samples containing 5 µM Aβ aggregates were prepared as indicated above. Dyes were added to the samples at a concentration of 5 µM for curcumin (Sigma-Aldrich #C1386) or 4 µM for hFTAA, and then samples were incubated for 15 min at room temperature with shaking (850 rpm). To remove unbound dye, samples were placed in Slide-A-Lyzer Mini dialysis devices with a molecular weight cutoff of 10 kDa (Thermo Scientific #69570) and dialysed against dH_2_O for ~ 50 min. After dialysis samples were recovered and placed in a half-area black clear-bottom 96-well microplate (Greiner Bio-One #675096). Fluorescence emission spectra were measured using a BMG CLARIOstar microplate reader. For curcumin, an excitation bandwidth of 432 ± 7.5 nm was used and fluorescence emission values from 460 ± 5 to 625 ± 5 nm were measured. For hFTAA, an excitation bandwidth of 488 ± 5 nm was used and fluorescence values from 513 ± 5 to 690 ± 5 nm were measured. Background signal from reactions containing only dye were subtracted, and then fluorescence signals were normalized to the highest value obtained, which was set at 1.0.

### Purification of brain-derived Aβ aggregates

PK-resistant Aβ aggregates were purified from the brain of an 8-month-old female TgCRND8 mouse [11] as previously described [73]. As a negative control, a brain from an 11-month-old male non-transgenic TgCRND8 littermate was subjected to the same purification protocol. For purification of Aβ aggregates from *App*^NL−F^ mice, brains from two 20-month-old female mice were used. The concentration of the purified Aβ aggregates was determined using an Aβ_1-x_ ELISA kit (IBL America #27729) following treatment with formic acid.

### Electron microscopy

Samples were sonicated in a water bath sonicator for 10 min prior to analysis. Negative-stain electron microscopy was performed as follows: 9 µL of purified brain-derived or recombinant PK-resistant Aβ aggregates were placed on a formvar/copper grid and then incubated for 2 min. Excess sample was removed using filter paper, and then 9 µL of 1% (w/v) phosphotungstic acid was added to the grid and incubated for 2 min. Excess phosphotungstic acid was removed and the grid was stored in the dark at room temperature until examined using either a Hitachi H7000 or a Talos L120C transmission electron microscope.

### Mice

Homozygous *App*^NL−F^ knock-in mice on a C57Bl/6 background [74], which express murine APP containing the Swedish (KM670/671NL) and Iberian/Beyreuther (I716F) mutations as well as a humanized Aβ region, were maintained on a 12 h light/12 h dark cycle and were given unlimited access to food and water. All studies utilized roughly equal numbers of male and female animals. All mouse experiments were performed in accordance with guidelines set by the Canadian Council on Animal Care under a protocol (AUP #4263.11) approved by the University Health Network Animal Care Committee.

### Intracerebral inoculations

Prior to inoculation, the purified or recombinant Aβ aggregates were diluted to a concentration of 33.3 ng/µL, sonicated for 10 min using a water bath sonicator, and then diluted 1:10 using inoculum diluent buffer [5% (w/v) BSA prepared in sterile PBS]. 6-week-old *App*^NL−F^ mice were anaesthetized using isoflurane gas and then freehand inoculated into the right parietal lobe at a depth of 3 mm with 30 µL of sample using a BD SafetyGlide 1 mL tuberculin syringe containing a 27-gauge 1/2″ needle (BD #305945). Each mouse received 100 ng of either purified or recombinant Aβ aggregates. As a negative control, dH_2_O diluted 1:10 (vol/vol) in inoculum diluent buffer was used. The individual performing the injections was not blinded to the identity of the inoculum. Inoculated mice were monitored daily for routine health. Mice were euthanized at 6 months post-inoculation (180–183 days post-inoculation) by transcardiac perfusion with 0.9% saline solution while under sodium pentobarbital anaesthesia (50 mg/kg). Brains were then removed from the skull and bisected parasagittally. The right half of the brain was fixed in 10% neutral buffered formalin and used for neuropathological analysis whereas the left half was frozen and stored at − 80 °C for biochemical studies.

### ELISAs

For determining total levels of Aβ42 in brain homogenates from inoculated mice, frozen hemibrains were homogenized to 10% (w/v) in sterile PBS using a Precellys MiniLys homogenizer and CK14 soft tissue homogenization kits (Bertin). Protein concentration was determined using the BCA assay (Thermo Scientific) and then 500 μg of total protein was brought up to a volume of 100 μL using PBS. Chilled 95% formic acid (200 µL; Sigma-Aldrich #F0507) was added to each sample, which were then sonicated in a water bath sonicator for 5 min. Samples were ultracentrifuged at 100,000 × *g* in a TLA-55 rotor for 1 h at 4 °C, and then the resulting supernatants were dried using a speed-vac. The dried proteins were resuspended in 50–100 μL of PBS**,** sonicated for 10 min using a Qsonica Q700 sonicator coupled to a microplate horn (#431MPX) set at 70% amplitude, and then stored at -80 °C. For analysis of PK-resistant Aβ42 levels, 500 μg of brain homogenate was digested with a final concentration of 100 μg/mL PK for 1 h at 37 °C with shaking in a volume of 100 μL (diluted with PBS; final PK:protein ratio of 1:50). The digestions were halted by addition of 2 mM PMSF, and then samples were treated with formic acid and processed identically to the undigested samples. For analysis of soluble Aβ42 levels, brain homogenates were treated with an equal volume of 0.4% (v/v) diethylamine/100 mM NaCl, ultracentrifuged at 100,000 × *g* for 1 h at 4 °C, and then neutralized by the addition of 0.1 volumes of 0.5 M Tris–HCl pH 6.8. Total, PK-resistant, and soluble Aβ42 levels were measured using Aβ_x-42_ ELISA kits (ThermoFisher Scientific #KHB3441) whereas levels of Aβ species containing an intact N-terminus were determined using an Aβ_1-x_ ELISA kit (IBL America #27729). Samples that fell below the lower detection limit for the Aβ_1-x_ ELISA and the soluble Aβ_x-42_ ELISA were not included in the analysis.

### Immunoblotting and silver staining

Nine volumes of 10% (w/v) brain homogenate were mixed with one volume of 10X detergent buffer [5% (v/v) NP-40, 5% (w/v) sodium deoxycholate in PBS] and then incubated on ice for 20 min. Samples were clarified by centrifugation at 1000 × *g* for 5 min at 4 °C to generate detergent-extracted brain homogenate. For analysis of insoluble Aβ, 0.5–1 mg of detergent-extracted brain homogenate was treated with a final concentration of 50 µg/mL PK in a volume of 100 µL for a final PK:protein ratio of 1:50. Digestions were performed for 1 h at 37 °C with shaking, and then reactions were halted by addition of PMSF to a final concentration of 2 mM. After the addition of sarkosyl to a final concentration of 2% (vol/vol), samples were ultracentrifuged at 48,000 rpm for 1 h at 4 °C using a TLA-55 rotor (Beckman Coulter). Pellets were resuspended in 1X Bolt LDS sample buffer containing 2.5% (vol/vol) β-mercaptoethanol, boiled, and then analyzed by immunoblotting. Samples were analyzed by SDS-PAGE using Bolt 4–12% Bis–Tris Plus gels (Thermo Scientific). For separation of individual Aβ variants, self-poured Bicine/Tris 10% polyacrylamide gels containing 8 M urea were used [82]. For silver staining, the Thermo Scientific Pierce Silver Stain Kit (catalog #PI24612) was used. For immunoblotting, proteins were transferred onto 0.45 μm Immobilon-P PVDF membranes and then membranes were blocked with 5% (w/v) non-fat skim milk in TBS containing 0.05% (v/v) Tween-20 (TBST). For the analysis of recombinant Aβ by immunoblotting, blots were boiled in PBS using a microwave prior to blocking. Membranes were incubated with anti-Aβ 6E10 antibody (BioLegend #803001; 1:4000 dilution) or anti-Aβ (N-terminal) antibody 82E1 (IBL America #10323; 1:2,000 dilution) diluted in blocking buffer overnight at 4 °C, and then blots were washed 3 times with TBST. Blots were then incubated with horseradish peroxidase-conjugated secondary antibodies (Bio-Rad) at room temperature followed by 3 washes with TBST. Blots were treated with Western Lightning ECL Pro (PerkinElmer) or SuperSignal West Dura ECL (Thermo Scientific) and then exposed to HyBlot CL film.

### Neuropathology

Formalin-fixed brains were embedded in paraffin and then processed for immunohistochemistry as previously described [44] using sagittal sections (5 µm) taken at the midline of the brain (~ 0.5–1 mm lateral) mounted onto glass slides. Sections were pre-treated with 88% formic acid for 6 min to facilitate detection of Aβ and then blocked using the M.O.M kit (Vector Laboratories). Immunostaining was performed using the following antibodies: anti-Aβ42 12F4 (BioLegend #805501; 1:2,000 dilution) or anti-Aβ (N-terminus) 82E1 (IBL America #10323; 1:1000 dilution). Sections were processed using the ImmPress HRP detection kit (Vector Laboratories), developed using 3,3′-diaminobenzidine (DAB), and counterstained with haematoxylin. Slides were either analyzed using a Leica DM6000B microscope and photographed using 20 × or 40 × objectives, or were scanned using the TissueScope LE120 slide scanner in conjunction with the TissueSnap preview station (Huron Digital Pathology). For semi-quantitative analysis of Aβ42 pathology in the brains of inoculated *App*^NL−F^ mice, the extent of pathology was scored across 4 different brain regions (occipital cortex, olfactory bulb, subcallosal region, and cerebellum) within a single section using the following system: 0, no Aβ deposition; 1, mild Aβ deposition; 2, moderate Aβ deposition; 3, intense Aβ deposition. Aβ CAA was assessed by counting the number of Aβ42-positive meningeal blood vessels overlying the frontal to occipital cortex. Spontaneous Aβ deposition was assessed by counting the number of Aβ plaques in the frontal/parietal cortex.

### Statistical analysis

All statistical analysis was performed using GraphPad Prism software (version 9.0.0) with a significance threshold of *P* = 0.05. For comparisons between groups of inoculated mice, Gaussian distributions were not assumed and therefore the Kruskal–Wallis test followed by Dunn’s multiple comparisons test was used. For in vitro samples, a standard one-way ANOVA followed by Tukey’s multiple comparisons test was used. For comparison between total and PK-resistant Aβ levels in Aβ43-inoculated mice, a paired, two-tailed *t*-test was used.

## Results

### Generation of recombinant Aβ aggregates for in vivo seeding studies

**Fig. 1:**
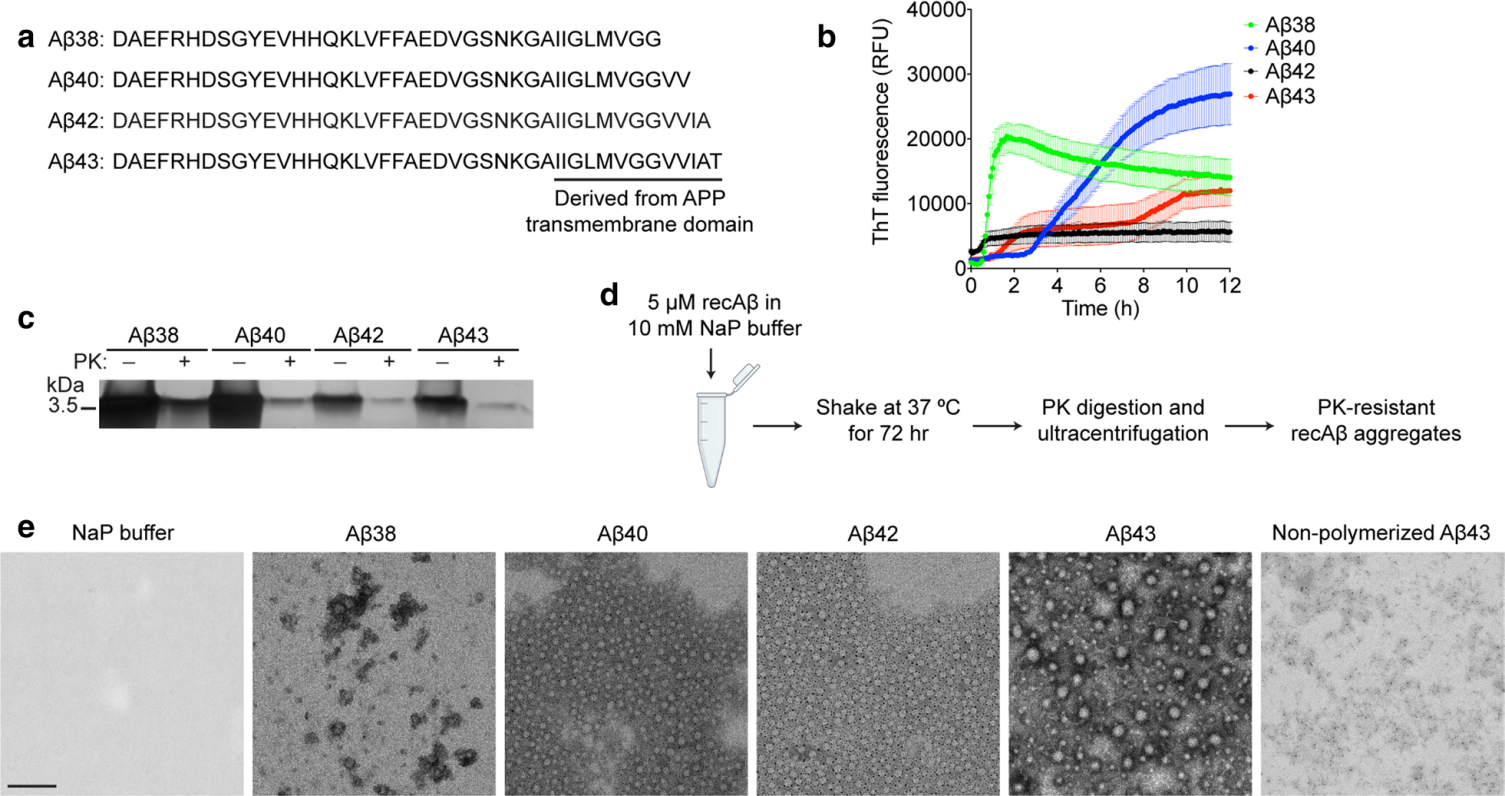
Generation of proteinase K-resistant recombinant Aβ aggregates. **a** Amino acid sequences of the recombinant Aβ38, Aβ40, Aβ42, and Aβ43 peptides. **b** Kinetics of recombinant Aβ38 (green), Aβ40 (blue), Aβ42 (black), and Aβ43 (red) aggregation in a real-time Thioflavin T (ThT) fluorescence assay. Data is mean ± s.e.m. for n = 5 independent biological replicates per Aβ variant. **c** Silver-stained SDS-PAGE gel of insoluble recombinant Aβ aggregates with (+) or without (−) digestion with proteinase K (PK). **d** Schematic of procedure used to generate PK-resistant recombinant Aβ aggregates for inoculation of *App*^NL−F^ mice. **e** Electron micrographs of the insoluble, PK-resistant recombinant Aβ aggregate preparations. As negative controls, sodium phosphate (NaP) buffer or non-polymerized Aβ43 were processed identically to the insoluble, PK-resistant Aβ preparations and then imaged. Scale bar = 100 nm (applies to all images)

The four most common full-length Aβ peptide variants are Aβ1-38, Aβ1-40, Aβ1-42, and Aβ1-43, which, for simplicity, will be referred to as Aβ38, Aβ40, Aβ42, and Aβ43, respectively. These peptides differ from each other at their C-termini, with the longer variants incorporating additional residues from the APP transmembrane domain (Fig. 1a). To characterize the relative seeding capacity of individual Aβ C-terminal variants, we generated Aβ aggregates by polymerizing recombinant Aβ peptides in sodium phosphate buffer by continuous shaking at 37 ºC. This buffer was chosen because it has previously led to the formation of proteinase K (PK)-resistant synthetic Aβ aggregates that exhibited seeding activity when injected into transgenic mice [83]. In a real-time Thioflavin T (ThT) fluorescence aggregation assay, all four Aβ peptides formed aggregates within 12 h as revealed by an increase in ThT fluorescence (Fig. 1b). As expected, a fraction of the polymerized Aβ preparations were resistant to PK digestion (Fig. 1c).

For inoculation studies, recombinant Aβ aggregates were generated by shaking at 37 ºC for three days followed by PK digestion and then ultracentrifugation to isolate insoluble, PK-resistant Aβ aggregates (Fig. 1d). When imaged by electron microscopy, the recombinant Aβ aggregates exhibited distinct morphologies. Whereas PK-resistant Aβ38 mainly consisted of amorphous aggregates, PK-resistant Aβ40 and Aβ42 preparations contained small, uniformly-sized spherical particles with a diameter of ~ 15 nm (Fig. 1e). In contrast, PK-resistant Aβ43 preparations consisted of larger spherical aggregates with diameters of ~ 25–30 nm. None of these structures were apparent in preparations containing only buffer or non-polymerized recombinant Aβ43 that were processed identically. Fibrils were rarely found in any of the PK-resistant recombinant Aβ preparations, indicating that the polymerization conditions utilized generate predominantly pre-fibrillar aggregates.

### Purification of brain-derived Aβ aggregates from AD transgenic mice

**Fig. 2:**
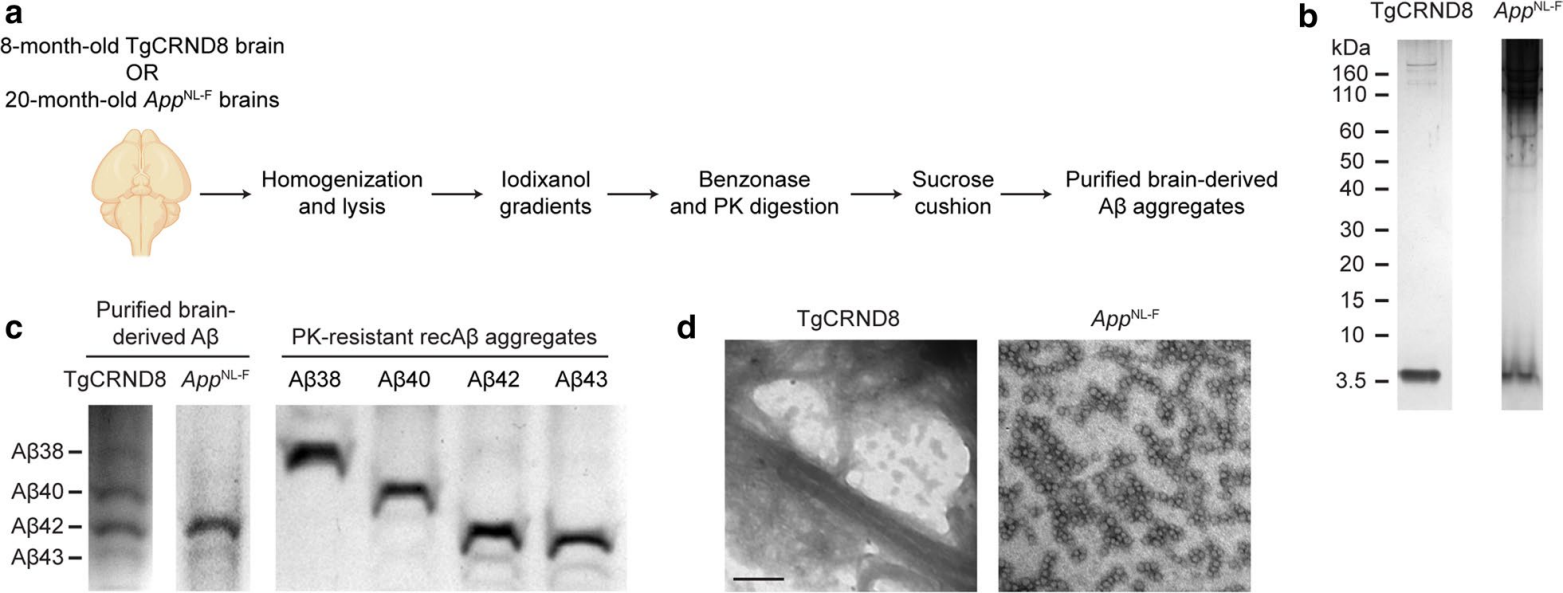
Purification of brain-derived Aβ aggregates from transgenic mice. **a** Schematic of the procedure used to purify PK-resistant Aβ aggregates from the brains of aged TgCRND8 and *App*^NL−F^ mice. **b** Silver-stained SDS-PAGE of the purified Aβ aggregates from TgCRND8 and *App*^NL−F^ mice. **c** Silver-stained urea gels of purified brain-derived and recombinant PK-resistant Aβ aggregates. **d** Electron micrographs of PK-resistant Aβ aggregates purified from the brains of TgCRND8 and *App*^NL−F^ mice. Scale bar = 100 nm (applies to both images)

For comparison purposes, we also purified Aβ aggregates from the brains of two AD mouse models, TgCRND8 and *App*^NL−F^, both of which develop prominent cerebral Aβ deposition as they age [11, 74]. To facilitate a direct comparison with recombinant Aβ aggregates, we employed a purification protocol that also involves PK digestion, and which produces Aβ seeds that are potent inducers of cerebral Aβ pathology (Fig. 2a) [73, 84]. In purified preparations from TgCRND8 mice, a band corresponding to the molecular weight of Aβ was the predominant species observed by SDS-PAGE followed by silver staining whereas additional high-molecular weight species were present in the preparations from *App*^NL−F^ mice (Fig. 2b). Using the PK-resistant recombinant Aβ aggregates as a reference, the Aβ variant composition of the purified TgCRND8 and *App*^NL−F^ Aβ preparations was analyzed by electrophoresis using urea-containing polyacrylamide gels [82]. All four Aβ peptides (Aβ38, Aβ40, Aβ42, and Aβ43) were present in the purified TgCRND8 Aβ preparation, although the relative levels of Aβ40 and Aβ42 were higher than Aβ38 and Aβ43 (Fig. 2c), similar to what we have previously determined [73]. In contrast, the purified Aβ fraction isolated from *App*^NL−F^ mice consisted predominantly of Aβ42 with trace amounts of Aβ43. Using electron microscopy, we observed that the purified, PK-resistant *App*^NL−F^ material was composed of Aβ protofibrils consisting of spherical particles arranged in linear clusters whereas the purified TgCRND8 material was mostly composed of plaques and fibrillar Aβ species (Fig. 2d).

### Inoculation of ***App***^NL−F^ mice with recombinant and brain-derived Aβ aggregates

**Fig. 3:**
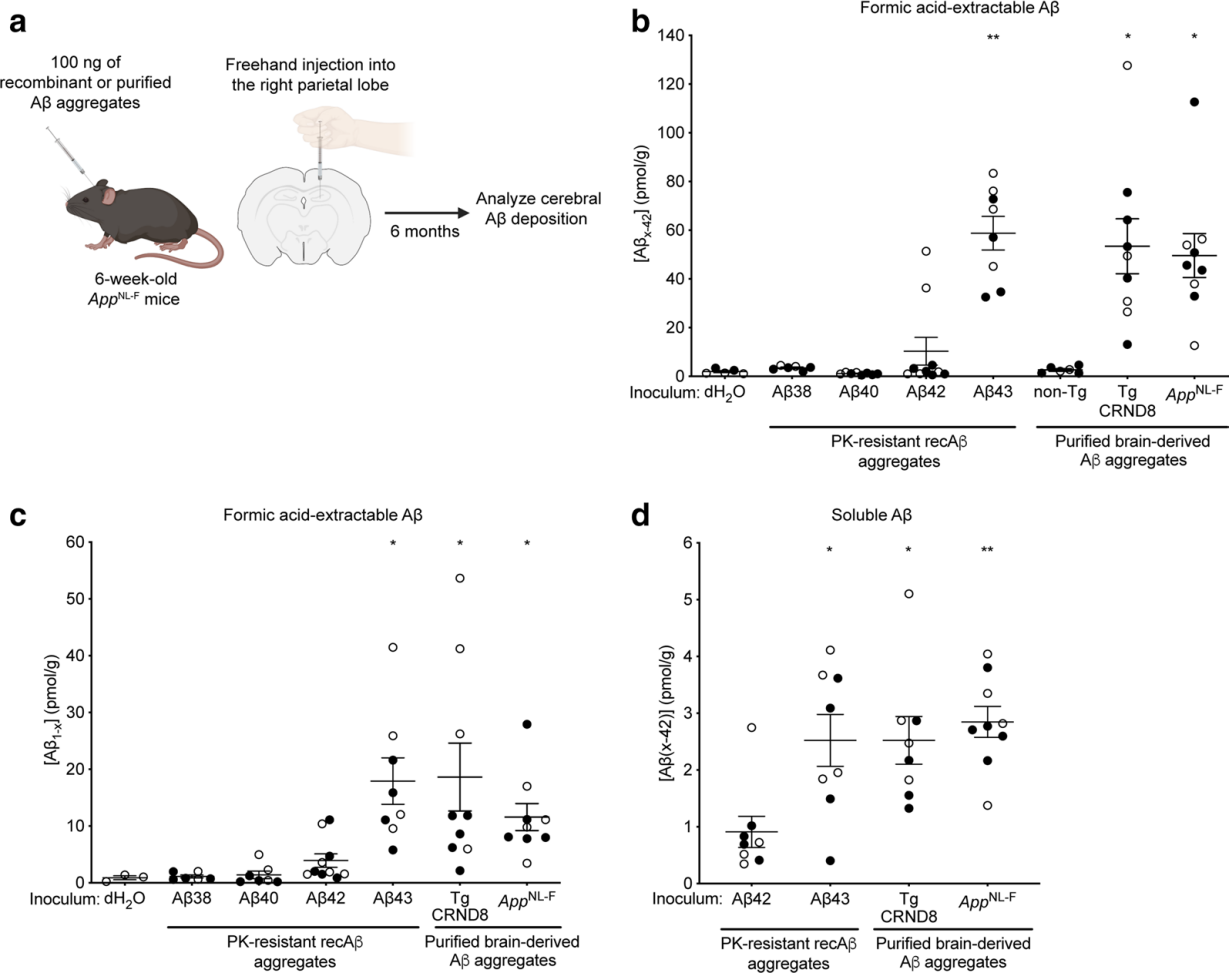
Increased cerebral Aβ42 levels in mice inoculated with recombinant Aβ43 aggregates. **a** Schematic of the intracerebral inoculation procedure in *App*^NL−F^ mice and the experimental timeline. **b** Formic acid-extractable Aβ42 levels (mean ± s.e.m.), as determined by Aβ_x-42_ ELISA, in brains from *App*^NL−F^ mice at 6 months post-inoculation with recombinant Aβ aggregates (n = 6, 8, 10, or 8 for Aβ38, Aβ40, Aβ42, and Aβ43, respectively) or purified brain-derived Aβ aggregates from either TgCRND8 (n = 9) or *App*^NL−F^ (n = 9) mice. Aβ42 levels in mice inoculated with either dH_2_O (n = 5) or material derived from a non-Tg mouse brain subjected to the Aβ purification protocol (n = 6) are also shown. Levels of Aβ42 were significantly higher in mice injected with recombinant Aβ43 aggregates or brain-derived Aβ aggregates compared to mice injected with dH_2_O (*P* = 0.0061 for Aβ43, *P* = 0.018 for TgCRND8, and *P* = 0.021 for *App*^NL−F^ as determined by a Kruskal–Wallis test followed by Dunn’s multiple comparisons test; all other groups are non-significant compared to dH_2_O-injected mice). **c** Levels of formic acid-extractable full-length total Aβ (mean ± s.e.m.), as determined by Aβ_1-x_ ELISA, in brains from *App*^NL−F^ mice inoculated with either dH_2_O (n = 3), Aβ38 (n = 6), Aβ40 (n = 7), Aβ42 (n = 10), Aβ43 (n = 8), TgCRND8 Aβ (n = 9), or *App*^NL−F^ Aβ (n = 9). Levels of total Aβ were significantly higher in mice injected with recombinant Aβ43 aggregates or brain-derived Aβ aggregates compared to mice injected with dH_2_O (*P* = 0.010 for Aβ43, *P* = 0.023 for TgCRND8, and *P* = 0.045 for *App*^NL−F^ as determined by a Kruskal–Wallis test followed by Dunn’s multiple comparisons test; all other groups non-significant compared to dH_2_O-injected mice). **d** Soluble Aβ42 levels (mean ± s.e.m.), as determined by Aβ_x-42_ ELISA, in brains from *App*^NL−F^ mice inoculated with recombinant Aβ42 aggregates (n = 8), recombinant Aβ43 aggregates (n = 8), TgCRND8 Aβ aggregates (n = 8), or *App*^NL−F^ Aβ aggregates (n = 9). Levels of soluble Aβ42 were significantly higher in mice injected with recombinant Aβ43 aggregates or brain-derived Aβ aggregates compared to mice injected with recombinant Aβ42 aggregates (*P* = 0.025 for Aβ43, *P* = 0.039 for TgCRND8, and *P* = 0.0039 for *App*^NL−F^ as determined by a Kruskal–Wallis test followed by Dunn’s multiple comparisons test). In panels b-d, open circles indicate female mice and filled circles indicate male mice

For the in vivo seeding studies, 6-week-old *App*^NL−F^ knock-in mice were intracerebrally injected with 100 ng of PK-resistant recombinant Aβ preparations or purified brain-derived Aβ aggregates (Fig. 3a). Prior to inoculation, an Aβ_1-x_ ELISA was used to measure the concentration of PK-resistant Aβ in each preparation to ensure that each mouse received an equal amount of Aβ aggregates. As negative controls, mice were injected with either dH_2_O or brain material from a non-transgenic TgCRND8 littermate, henceforth referred to as non-Tg, that was subjected to the same purification protocol. Mice were injected into the right cerebral hemisphere using a freehand inoculation technique previously shown to be an effective means of introducing Aβ seeds into the brain and which results in similar kinetics of induced Aβ accumulation to mice injected using a stereotactic technique [73, 84, 93, 95]. With the freehand technique, the Aβ seeds are predominantly delivered into the hippocampus and overlying cortical regions, but it is likely that a portion of the seeds enter the ventricles as well. Mice were euthanized at 6 months post-inoculation (~ 7.5 months of age), a timepoint at which minimal spontaneous Aβ deposition is present in the brain [73]. Both male and female mice were analyzed since it has been shown that the kinetics of spontaneous Aβ accumulation are accelerated in female AD mouse models [8]. As expected, all Aβ-inoculated mice remained free of overt signs of neurological illness for the duration of the experiment, although a subset of mice died of intercurrent illness (Additional file 1: Table S1) and were excluded from analysis.

Analysis of total (formic acid-extractable) Aβ levels in brain homogenates from the groups of inoculated mice using an ELISA specific for Aβ species ending at residue 42 (Aβ_x-42_) revealed significant differences (Fig. 3b). Levels of cerebral Aβ42 in mice injected with Aβ38 or Aβ40 aggregates were identical to those in mice injected with dH_2_O or the non-Tg mock-purified sample, suggesting that no induction of Aβ deposition occurred in these mice. In contrast, elevated Aβ42 levels were present in 20% (2 of 10) Aβ42-injected mice and 100% (8 of 8) Aβ43-injected mice. Remarkably, cerebral Aβ42 levels in mice injected with Aβ43 aggregates were similar to those in mice injected with purified brain-derived aggregates from either TgCRND8 mice or *App*^NL−F^ mice (Fig. 3b). There was no consistent difference in Aβ42 levels between male and female Aβ-inoculated mice, suggesting that the presence of Aβ seeds may override sex-specific differences in the kinetics of spontaneous Aβ deposition, as has been noted previously [73]. Similar results were obtained when an ELISA that recognizes all Aβ variants with an intact N-terminus (Aβ_1-x_) was used (Fig. 3c), suggesting that Aβ42 is the predominant Aβ species induced in the inoculated *App*^NL−F^ mice. The lower absolute levels of Aβ when measured using the Aβ_1-x_ assay may reflect differential sensitivities and capture efficiencies between the two ELISAs but could also suggest that a portion of the induced Aβ42 species may be N-terminally truncated [41].

**Fig. 4:**
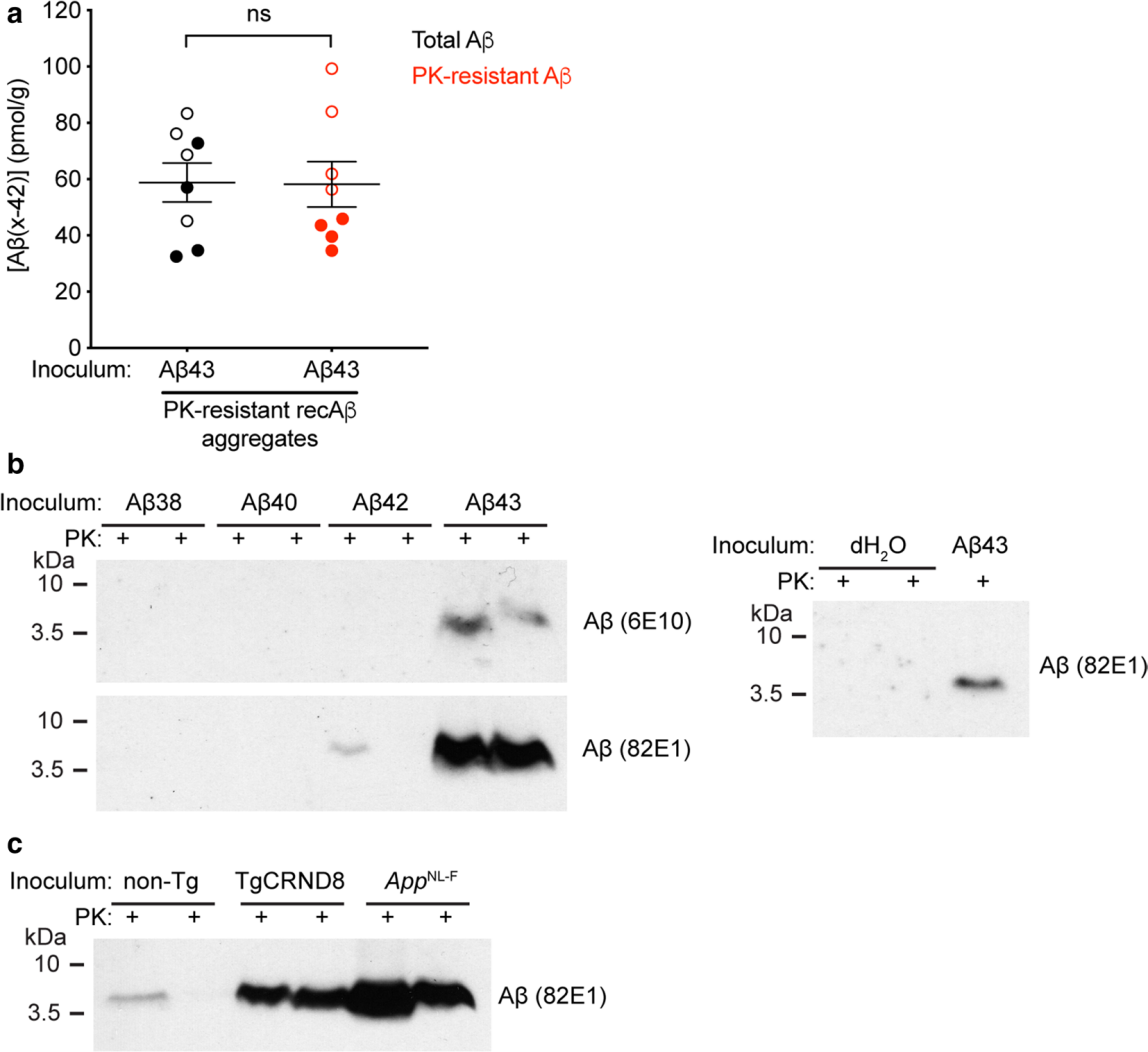
Induction of protease-resistant Aβ species in the brains of Aβ43-inoculated *App*^NL−F^ mice. **a** Levels of PK-resistant Aβ42 were not significantly different from levels of total Aβ42 in mice injected with recombinant Aβ43 aggregates (n = 8; *P* = 0.91 by a paired two-tailed *t* test). Open circles indicate female mice and filled circles indicate male mice. **b** Immunoblot of insoluble, PK-resistant Aβ species in brain homogenates from *App*^NL−F^ mice inoculated with either dH_2_O or PK-resistant recombinant Aβ38, Aβ40, Aβ42, or Aβ43 aggregates. Aβ was detected using the antibodies 6E10 or 82E1. **c** Immunoblot of insoluble, PK-resistant Aβ species in brain homogenates from *App*^NL−F^ mice inoculated with either non-Tg sample, TgCRND8 Aβ aggregates, or *App*^NL−F^ Aβ aggregates. Aβ was detected using the antibody 82E1

While levels of soluble Aβ42 in mice inoculated with recombinant Aβ43 aggregates or brain-derived Aβ aggregates were ~ 2.5-fold higher than in mice injected with recombinant Aβ42 aggregates (Fig. 3d), soluble Aβ42 levels were ~ 25-fold lower than total Aβ levels in Aβ43-injected animals. Moreover, levels of PK-resistant Aβ42 in Aβ43-inoculated mice were not significantly different from total Aβ42 levels, as determined by ELISA (Fig. 4a). These results imply that the majority of cerebral Aβ42 species in mice injected with Aβ43 are present in an aggregated state. To further confirm the presence of Aβ aggregates, we looked for the presence of detergent-insoluble, PK-resistant Aβ species in brain homogenates from inoculated *App*^NL−F^ mice. All of the mice inoculated with Aβ43 aggregates exhibited PK-resistant Aβ species in their brains, whereas PK-resistant Aβ was detected in only 2 of 10 Aβ42-inoculated animals (Additional file 2: Fig. S1). In brain homogenates from Aβ43-injected mice, PK-resistant Aβ could be detected with two distinct Aβ antibodies (Fig. 4b). Brain homogenates from mice injected with brain-derived Aβ aggregates from either TgCRND8 or *App*^NL−F^ mice also contained PK-resistant Aβ species (Fig. 4c).

### Neuropathological analysis of Aβ-inoculated ***App***^NL−F^ mice

**Fig. 5:**
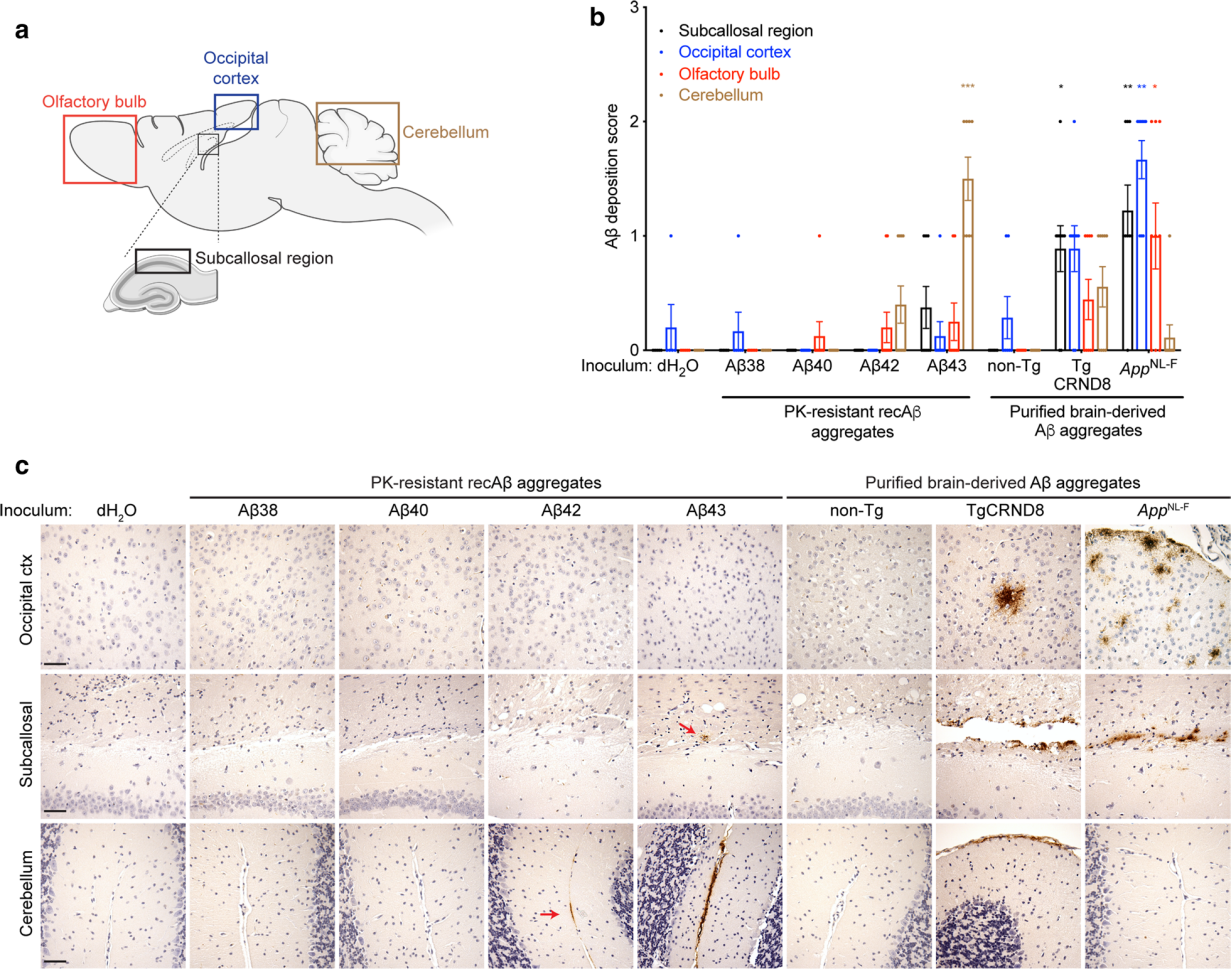
Induced Aβ42 deposition in *App*^NL−F^ mice inoculated with recombinant Aβ43 aggregates. **a** Schematic of the locations of induced Aβ pathology in the brains of *App*^NL−F^ mice. **b** Semi-quantitative scoring of Aβ42 pathology (12F4 immunohistochemistry) in mice at 6 months post-inoculation with recombinant Aβ aggregates (n = 6, 8, 10, or 8 for Aβ38, Aβ40, Aβ42, and Aβ43, respectively) or purified brain-derived Aβ aggregates from either TgCRND8 (n = 9) or *App*^NL−F^ (n = 9) mice. Mice inoculated with either dH_2_O (n = 5) or material derived from a non-Tg mouse brain (n = 6) were used as negative controls. Compared to mice injected with dH_2_O, there was a significant increase in Aβ42 pathology in the cerebellum of mice injected with Aβ43 aggregates (*P* = 0.00029), in the occipital cortex of mice inoculated with *App*^NL−F^ Aβ aggregates (*P* = 0.0039), in the olfactory bulb of mice inoculated with *App*^NL−F^ Aβ aggregates (*P* = 0.022), and in the subcallosal region of mice inoculated with *App*^NL−F^ (*P* = 0.0022) or TgCRND8 (*P* = 0.022) Aβ aggregates, as determined by a Kruskal–Wallis test followed by Dunn’s multiple comparisons test. **c** Representative images of Aβ42 pathology (12F4 immunohistochemistry) in the occipital cortex, subcallosal region, and cerebellum of *App*^NL−F^ mice at 6 months post-inoculation with the indicated Aβ preparations. Red arrows indicate minor amounts of Aβ deposition in the subcallosal region of Aβ43-inoculated mice and the cerebellum of Aβ42-inoculated mice. Scale bars = 50 µm (applies to all images)

Small numbers of Aβ42 deposits were observed in the frontal and parietal cortices of all groups of control- and Aβ-inoculated *App*^NL−F^ mice, likely indicative of a minimal amount of spontaneous Aβ pathology in mice of this age (Additional file 3: Fig. S2). In contrast, all mice inoculated with recombinant Aβ43 aggregates exhibited prominent induced Aβ42 deposition in the cerebellum (Fig. 5a–c). A subset of Aβ42-inoculated mice (4 of 10) also exhibited cerebellar Aβ pathology, but of a lower intensity than that seen in Aβ43-injected animals. A minor amount of induced Aβ42 deposition was also observed in the subcallosal region of a subset of Aβ43-inoculated *App*^NL−F^ mice (3 of 8), which was not found in any of the Aβ42-inoculated mice (Fig. 5b, c). Similar results were obtained when staining with an antibody that recognizes the N-terminus of Aβ (Additional file 4: Fig. S3). No significant Aβ42 pathology was observed in the brains of mice inoculated with recombinant Aβ38 or Aβ40 aggregates. Taken together, these results indicate that Aβ43 aggregates exhibit the highest propensity for inducing Aβ42 deposition in *App*^NL−F^ mice.

Induced Aβ42 deposition was also found in the brains of *App*^NL−F^ mice injected with purified Aβ aggregates derived from the brains of either TgCRND8 or *App*^NL−F^ mice when compared to mice injected with the non-Tg sample (Fig. 5b, c). However, unlike the robust Aβ42 deposition observed in the cerebellum of mice injected with recombinant Aβ43 aggregates, comparatively minor amounts of cerebellar Aβ42 deposition were observed in only 5 of 9 mice injected with TgCRND8-derived Aβ aggregates and only 1 of 9 mice injected with *App*^NL−F^-derived Aβ. Instead, the induced Aβ42 pathology in mice injected with brain-derived Aβ aggregates was prominently located in the subcallosal region, similar to what we have previously described [73], as well as the occipital cortex (Fig. 5b, c). A similar pattern was observed when sections from mice inoculated with brain-derived Aβ aggregates were stained with an N-terminal Aβ antibody (Additional file 4: Fig. S3).

**Fig. 6:**
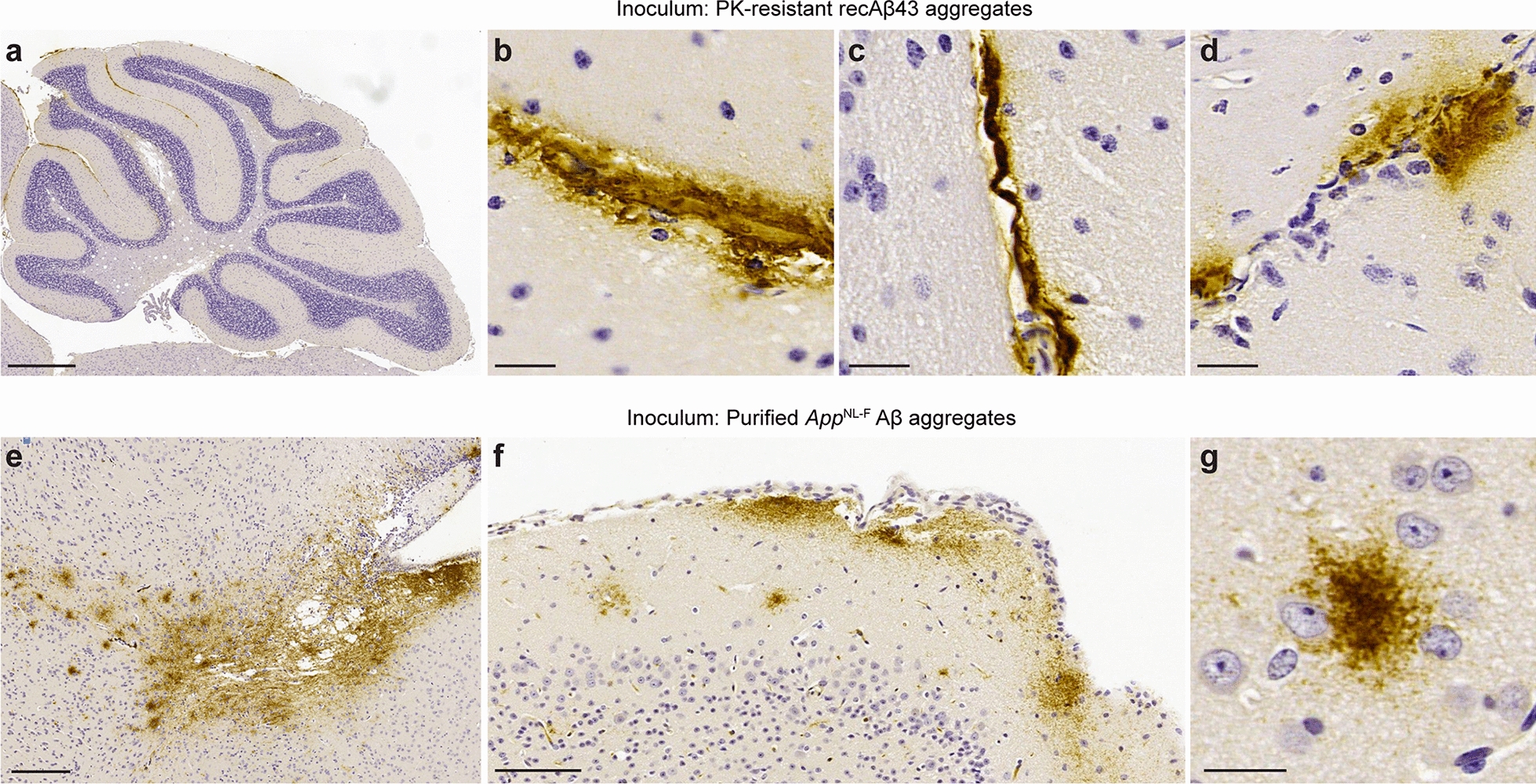
Distinct types of Aβ42 pathology in *App*^NL−F^ mice inoculated with recombinant Aβ43 aggregates or purified *App*^NL−F^ Aβ aggregates. **a** Cerebellar Aβ42 deposition (12F4 immunostaining) in an *App*^NL−F^ mouse at 6 months post-inoculation with recombinant Aβ43 aggregates. **b–d** Leptomeningeal Aβ42 deposition in Aβ43-inoculated mice at the interface between cerebellar folds (**b**), at the interface between the cerebellum and the midbrain (**c**), and at the interface between the cortex and the midbrain (**d**). **e** Spreading of Aβ42 pathology into the frontal cortex of an *App*^NL−F^ mouse at 6 months post-inoculation with purified *App*^NL−F^ Aβ aggregates. **f** Spreading of Aβ42 pathology into the occipital cortex of a mouse inoculated with purified *App*^NL−F^ Aβ aggregates. **g** Diffuse Aβ42 plaque in the occipital cortex of a mouse inoculated with purified *App*^NL−F^ Aβ aggregates. Scale bars = 500 µm (**a**), 20 µm (**b**–**d**, **g**), 200 µm (**e**), or 100 µm (**f**)

The induced Aβ42 pathology in the cerebellum of *App*^NL−F^ mice injected with recombinant Aβ43 aggregates was confined to the leptomeninges (Fig. 6a). Leptomeningeal Aβ42 deposition was observed in between the cerebellar folds (Fig. 6b) and at the interface between the cerebellum and the midbrain (Fig. 6c). Induced Aβ42 pathology was also observed at the interface between the cortex and the midbrain (Fig. 6d). In all instances of leptomeningeal Aβ42 deposition in Aβ43-inoculated mice, there was minimal to no spread of the Aβ aggregates into the parenchyma. In contrast, there was robust spread of induced Aβ42 pathology into the frontal and occipital cortex of *App*^NL−F^ mice inoculated with purified *App*^NL−F^ Aβ aggregates (Fig. 6e, f). The cortical Aβ42-containing plaques in the brains of mice inoculated with purified brain-derived Aβ aggregates were largely diffuse in nature (Fig. 6g).

**Fig. 7:**
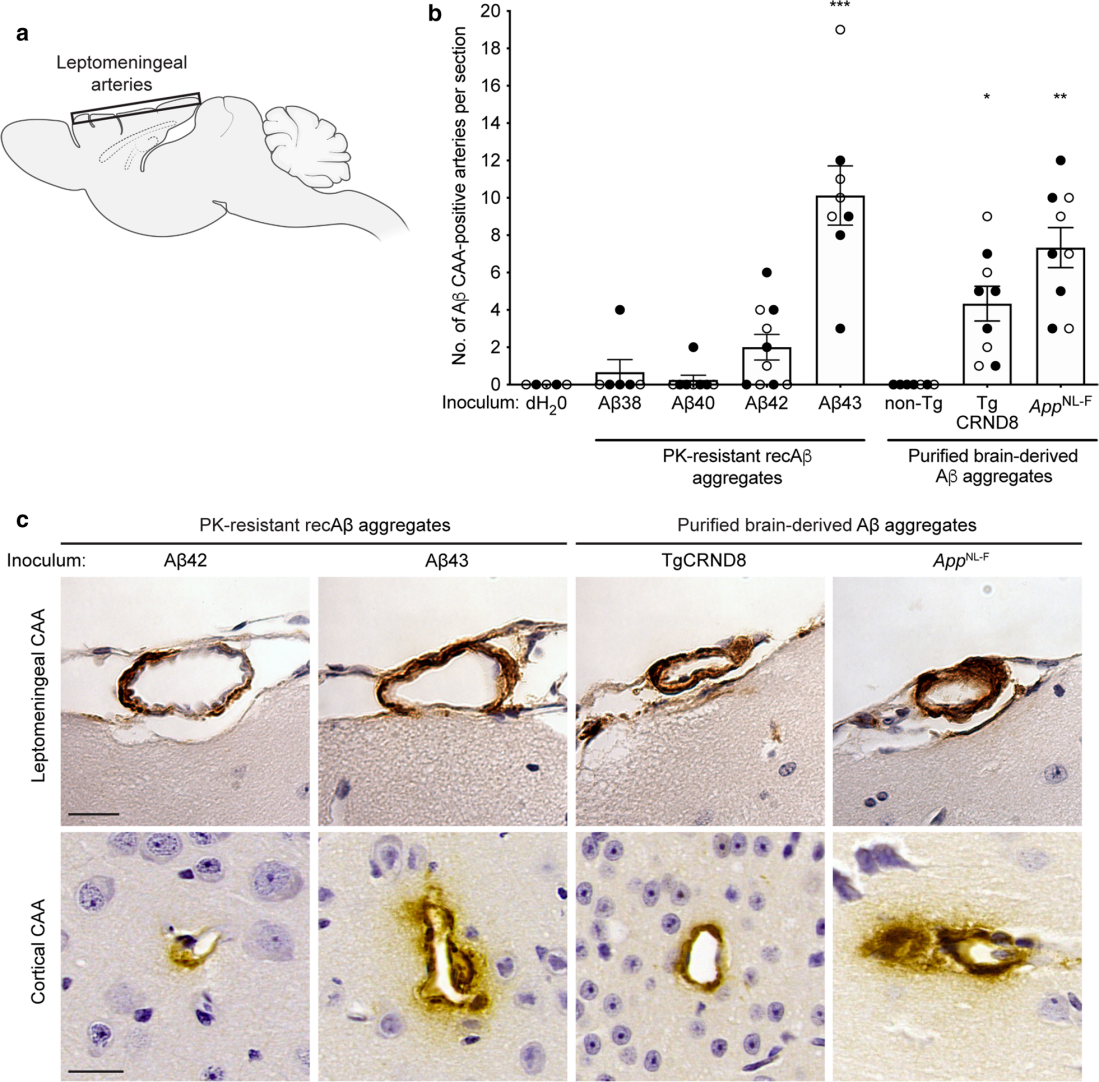
Induction of Aβ CAA in Aβ43-inoculated *App*^NL−F^ mice. **a** Schematic of the location of Aβ CAA in the brains of inoculated *App*^NL−F^ mice. **b** Quantification of Aβ42 CAA-positive leptomeningeal arteries (12F4 immunostaining) in *App*^NL−F^ mice at 6 months post-inoculation with either Aβ38 (n = 6), Aβ40 (n = 8), Aβ42 (n = 10), Aβ43 (n = 8), TgCRND8 (n = 9), or *App*^NL−F^ Aβ (n = 9). Mice inoculated with either dH_2_O (n = 5) or material derived from a non-Tg mouse brain (n = 6) were used as negative controls. The extent of Aβ CAA was significantly higher in mice injected with recombinant Aβ43 aggregates or brain-derived Aβ aggregates compared to mice injected with dH_2_O (*P* = 0.00040 for Aβ43, *P* = 0.043 for TgCRND8, and *P* = 0.0019 for *App*^NL−F^ as determined by a Kruskal–Wallis test followed by Dunn’s multiple comparisons test; all other groups non-significant compared to dH_2_O-injected mice). Open circles indicate female animals and filled circles indicate male animals. **c** Representative images of leptomeningeal (top row) and cortical (bottom row) Aβ42 CAA (12F4 immunostaining) in *App*^NL−F^ mice at 6 months post-inoculation with the indicated Aβ preparations. Scale bar = 20 µm (applies to all images)

In line with our prior findings in Aβ-inoculated *App*^NL−F^ mice [73], prominent Aβ42-containing CAA in the leptomeningeal arteries was also observed in all mice inoculated with Aβ43, TgCRND8 Aβ, or *App*^NL−F^ Aβ aggregates (Fig. 7a–c). In mice injected with Aβ42 aggregates, moderate leptomeningeal Aβ42 CAA was present in 60% of the animals, whereas only one mouse each in the groups inoculated with either Aβ38 or Aβ40 aggregates exhibited detectable CAA (Fig. 7b, c). Cortical Aβ42 CAA was also observed in mice injected with Aβ42, Aβ43, TgCRND8 Aβ, or *App*^NL−F^ Aβ aggregates (Fig. 7c), but this was much less prominent than the leptomeningeal CAA. None of the control-inoculated mice exhibited any Aβ42-containing leptomeningeal CAA, suggesting that spontaneous Aβ CAA is not common in *App*^NL−F^ mice at this age. While Aβ40 is the principal component of CAA in AD [9], the presence of Aβ42 in the leptomeningeal blood vessels of Aβ-inoculated *App*^NL−F^ mice likely reflects the presence of the Iberian/Beyreuther *APP* mutation, which results in a large increase in the Aβ42:Aβ40 ratio [22, 51].

### Conformational analysis of recombinant Aβ aggregates

**Fig. 8:**
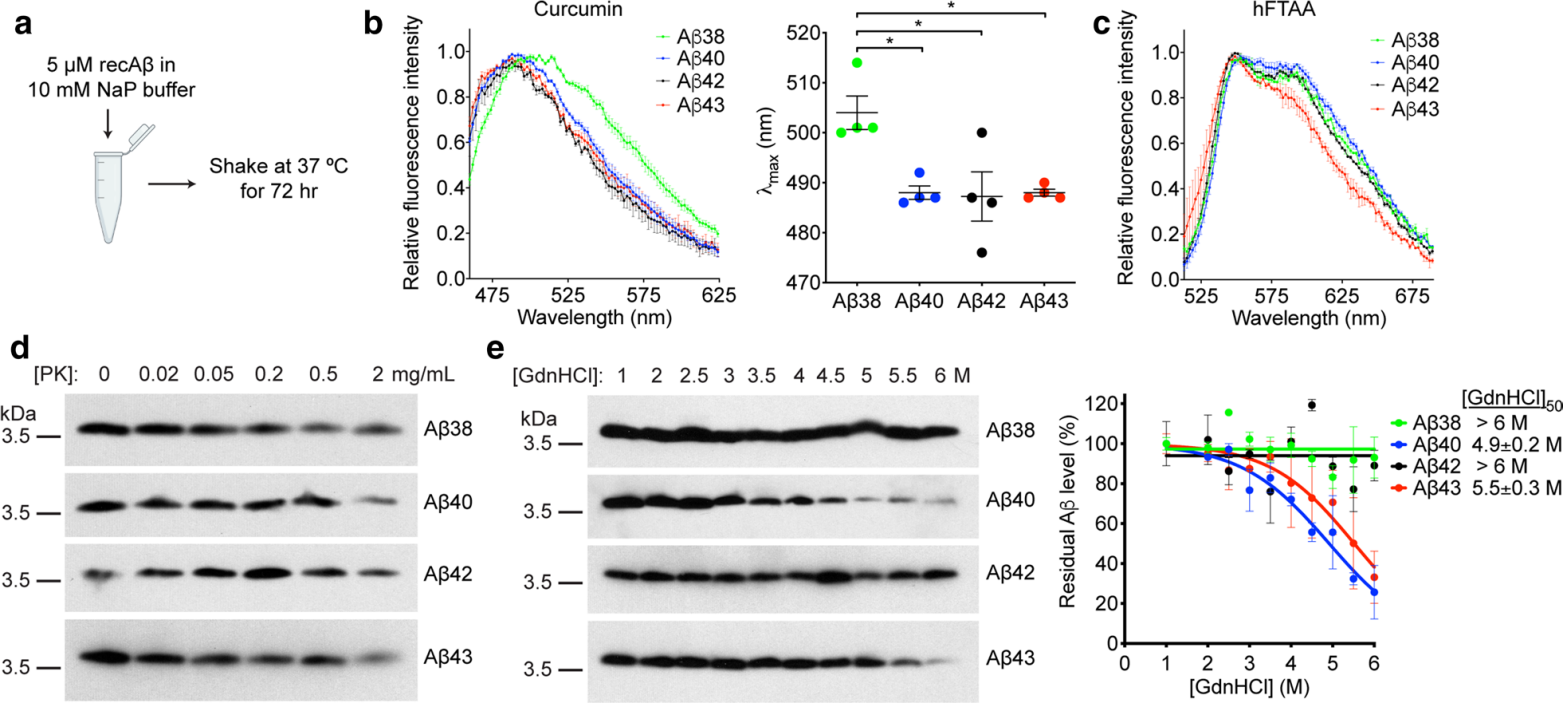
Conformational characterization of recombinant Aβ aggregates. **a** Schematic of the formation conditions for recombinant Aβ aggregates. **b** Fluorescence emission spectra (left panel) for curcumin bound to recombinant Aβ38 (green), Aβ40 (blue), Aβ42 (black), and Aβ43 (red) aggregates. The curcumin λ_max_ values (right panel) for Aβ38 aggregates were significantly higher than for the other Aβ variants (*P* = 0.016 vs. Aβ40, *P* = 0.014 vs. Aβ42, and *P* = 0.016 vs. Aβ43 by one-way ANOVA followed by Tukey’s multiple comparisons test). Data is mean ± s.e.m for 4 biologically independent aggregate preparations. **c** Fluorescence emission spectra for hFTAA bound to recombinant Aβ38 (green), Aβ40 (blue), Aβ42 (black), and Aβ43 (red) aggregates. Data is mean ± s.e.m for 2–3 biologically independent aggregate preparations. **d** Immunoblots (6E10 antibody) of the insoluble fraction following exposure of recombinant Aβ aggregates to the indicated concentrations of proteinase K (PK). **e** Conformational stability assays for recombinant Aβ aggregates. Representative Aβ immunoblots (left panel; 6E10 antibody) and the resultant denaturation curves (right panel) are shown. The curves depict mean residual insoluble Aβ values ± s.e.m. following treatment with the indicated concentrations of GdnHCl, and the calculated [GdnHCl]_50_ values are shown. n = 3 biologically independent aggregate preparations per Aβ variant

Given the markedly different in vivo seeding activities observed between Aβ aggregates composed of different Aβ C-terminal variants, we asked whether this may be due in part to conformational differences among the aggregates. For these studies, we generated recombinant Aβ aggregates as before but we did not isolate the PK-resistant fraction prior to analysis (Fig. 8a). We first assessed fluorescence emission spectra upon binding of the conformation-sensitive dyes curcumin and heptamer-formyl thiophene acetic acid (hFTAA) to the various Aβ aggregates [13, 36, 61, 70]. While the curcumin emission spectra for Aβ40, Aβ42, and Aβ43 aggregates were essentially superimposable, the curve for Aβ38 aggregates was red-shifted, with a significantly higher λ_max_ value (Fig. 8b). With hFTAA, the emission spectrum for Aβ43 aggregates differed from the other three, with a reduced second peak around 600 nm (Fig. 8c). All of the Aβ aggregates were highly resistant to PK digestion, even up to concentrations of 2 mg/mL PK (Fig. 8d). To further characterize the conformational properties of the recombinant Aβ aggregates, we performed conformational stability assays, which measure the relative resistance of the aggregates to denaturation with guanidine hydrochloride [46]. While none of the aggregates was fully solubilized by 6 M guanidine hydrochloride, Aβ40 and Aβ43 aggregates were significantly less stable than either Aβ38 or Aβ42 aggregates (Fig. 7e). Collectively, these results suggest that while all four Aβ C-terminal variants form highly PK-resistant aggregates, conformational differences may exist among them.

## Discussion

In this study, we investigated the relative prion-like seeding capacities of individual Aβ C-terminal variants in the *App*^NL−F^ AD mouse model using aggregates composed of recombinant Aβ. Given that *App*^NL−F^ mice predominantly produce Aβ42 [74] and that nucleation-dependent polymerization is generally most efficient when the seed and substrate are composed of identical protein species, we had predicted that recombinant Aβ42 aggregates would exhibit higher seeding propensity than Aβ38, Aβ40, or Aβ43 aggregates. Instead, we found that Aβ43 aggregates were the most potent seeds and were as effective as brain-derived Aβ aggregates at inducing the accumulation and deposition of Aβ42 in the brain. A subset of mice inoculated with Aβ42 aggregates also exhibited some induced Aβ42 deposition and pathology, suggesting the existence of a “seeding capacity gradient” in which Aβ43 would be the peptide with the highest seeding capacity followed by Aβ42, with Aβ40 and Aβ38 falling in the ineffectual range. It is noteworthy that the purified brain-derived Aβ aggregates from TgCRND8 and *App*^NL−F^ mice both contained detectable amounts of Aβ43. While it is not possible at this time to ascribe the seeding activity present in these samples to Aβ43, the lack of detectable Aβ40 and Aβ38 in the purified *App*^NL−F^ aggregates and the comparable seeding activity of *App*^NL−F^ Aβ aggregates to TgCRND8 Aβ aggregates suggests that longer Aβ variants are more important for the observed seeding behavior than shorter variants.

Previous studies revealed that synthetic Aβ40 and Aβ42 aggregates are capable of inducing cerebral Aβ pathology in a transgenic AD mouse model [83, 84], whereas we did not observe any seeding activity with recombinant Aβ40 aggregates and only marginal activity with Aβ42 aggregates. The simplest explanation for this discrepancy relates to the amount of Aβ aggregates injected into the mice. In our study, mice received 100 ng of recombinant Aβ aggregates whereas in the previous studies mice received 7.5 or 12 µg of Aβ, a 75- to 120-fold difference. Indeed, the efficiency of Aβ pathology induction in mice is known to be directly proportional to the amount of seed material injected [56]. Furthermore, in the previous studies, the induction of cerebral Aβ deposition was assessed at 11 months post-inoculation whereas we analyzed the Aβ-injected mice at 6 months post-inoculation to minimize the co-occurrence of spontaneous Aβ pathology. This extra time may have permitted the amplification and propagation of Aβ seeds that were initially in low abundance. Finally, it should be noted that a different AD mouse model was used in the aforementioned studies. APP23 transgenic mice express APP containing only the Swedish mutation and thus, at all ages, produce more Aβ40 than Aβ42 [85, 97], which might render the mice more susceptible to Aβ40 seeds.

Inoculation of *App*^NL−F^ mice with recombinant Aβ43 aggregates resulted in a similar amount of cerebral Aβ42 accumulation as in mice injected with identical quantities of brain-derived Aβ aggregates, suggesting that Aβ43 facilitates the in vitro generation of Aβ aggregates with seeding activities comparable to brain-derived material. Previous studies had determined that synthetic Aβ40 aggregates were approximately 100-fold less potent than brain-derived Aβ aggregates at inducing Aβ pathology in the mouse brain [84]. A potential explanation for this difference is that synthetic and brain-derived Aβ40 aggregates are structurally distinct and thus constitute distinct Aβ strains [38, 45]. We speculate that recombinant Aβ43 aggregates adopt a structure that is more similar to brain-derived Aβ aggregates than can be obtained by polymerization of Aβ40 or Aβ42 in vitro. However, the structures of recombinant Aβ43 and brain-derived Aβ aggregates must not be identical, as the two types of Aβ assemblies produced distinct neuropathological signatures upon inoculation in mice and thus may comprise unique strains. In particular, prominent cerebellar Aβ deposition within the leptomeninges was observed in mice injected with recombinant Aβ43 aggregates. Interestingly, this pattern resembled that observed when *App*^NL−F^ mice were injected with archival batches of cadaveric human growth hormone that produced a CAA-dominant Aβ pathology when administered to humans [68]. Since CAA was also a striking feature in Aβ43-inoculated mice, the structure of recombinant Aβ43 aggregates may share some characteristics with the Aβ seeds present in the growth hormone preparations.

While a molecular explanation for the differential distribution of Aβ pathology induced by recombinant Aβ43 and brain-derived Aβ aggregates remains to be determined, we hypothesize that it may be related to the relative ability of the aggregates to migrate into the parenchyma. Using the freehand inoculation technique, it is likely that a portion of the Aβ seeds were introduced into the ventricles, resulting in their widespread distribution throughout the brain via CSF circulation pathways. Brain-derived Aβ seeds may be better at templating the production of Aβ aggregates that are capable of entering the parenchyma, which is consistent with our observation that the majority of induced parenchymal Aβ pathology in mice injected with brain-derived seeds was found in proximity to the surface of the brain or in the vicinity of the ventricular system. In contrast, the Aβ pathology induced by recombinant Aβ43 seeds may remain confined to the leptomeninges because they are unable to spread into the parenchyma. The reason for this differential spread may be related to the size of the induced Aβ aggregates [43] or the differential affinity of recombinant and brain-derived Aβ aggregates for putative Aβ receptors that may be required for transit from the leptomeninges into the parenchyma [35, 47].

We envision two possible explanations for the differential seeding activities observed for aggregates composed of distinct Aβ C-terminal variants, although these are not mutually exclusive. First, Aβ43 aggregates may consist of a unique structure that exhibits a higher propensity for self-propagation in vivo. Under the polymerization conditions we employed, the PK-resistant Aβ43 aggregates adopted a pre-fibrillar structure, which was distinct from those present in the Aβ38, Aβ40, and Aβ42 preparations as well as the protofibrils and fibrils present in the brain-derived Aβ preparations. We also note that recombinant Aβ43 aggregates exhibited a distinct spectral signature when bound to hFTAA, arguing for structural variances among the different aggregate preparations. A second possibility is that Aβ43 aggregates are either more or less stable upon injection into mice than the other Aβ aggregates. In prion disease, less stable prion strains replicate more quickly in animals, and Aβ aggregates with lower stability appear to propagate more rapidly in mice [50, 94]. Since the Aβ43 aggregates were more susceptible to denaturation with guanidine hydrochloride than Aβ42 aggregates, they may be more frangible and thus generate a greater quantity of seeds when injected into mice. On the other hand, it is plausible that exogenous Aβ43 aggregates may be cleared at a slower rate in vivo, which may permit prolonged exposure to Aβ seeds and therefore increased Aβ propagation.

We do not know whether the enhanced seeding activity of the Aβ43 aggregates was due to the specific Aβ assembly state formed using these conditions or whether multiple types of Aβ43 assemblies (oligomers, protofibrils, fibrils, etc.) all exhibit heightened prion-like seeding behavior. A limitation of our study is that we only used a single set of conditions to generate the recombinant Aβ aggregates. It is widely documented that varying the buffer and polymerization conditions can lead to the formation of structurally distinct Aβ “polymorphs” [37, 53, 66]. Indeed, while we polymerized Aβ43 at a concentration of 5 μM in sodium phosphate buffer to obtain PK-resistant pre-fibrillar aggregates [83], polymerization of Aβ43 at a concentration of 10 μM in phosphate-buffered saline resulted in the generation of Aβ43 fibrils [10]. It will also be important to investigate the seeding activity of recombinant Aβ43 aggregates in other APP mouse models, including those that lack the Iberian/Beyreuther mutation and thus produce an ensemble of Aβ C-terminal variants that better resembles that observed in sporadic/late-onset AD.

To date, a majority of studies have focused on the two most abundant variants of Aβ, Aβ40 and Aβ42, with the evidence pointing to Aβ42 as the key mediator of AD pathogenesis since it is more prone to aggregate into neurotoxic species [29, 42] and its levels are selectively increased by AD-causing mutations in the presenilin genes [12, 16, 78]. However, a potential important role for Aβ43 in AD is becoming increasingly recognized. Unlike Aβ42, which is derived from Aβ48 via Aβ45 by sequential presenilin cleavage, Aβ43 is generated from Aβ49 via Aβ46 [86]. Once Aβ43-specific antibodies became available, it was discovered that Aβ43 levels are increased and Aβ43 deposits are abundant in AD and Down syndrome brains, despite low absolute amounts of the peptide relative to Aβ40 and Aβ42 [26, 27, 65, 77, 96]. Moreover, like Aβ42, Aβ43 readily forms aggregates in vitro and in vivo that are neurotoxic [5, 7, 14, 54, 76, 79], and lower levels of Aβ43 in the cerebrospinal fluid of AD patients seems to be strongly correlated with cerebral Aβ deposition in the same way as lower levels of Aβ42 [1, 48].

While it is conceivable that N-terminal modifications in Aβ such as truncation and pyroglutamylation at residue 3 may further modulate seeding activity [60], our data suggests that Aβ43 aggregates with an intact N-terminus possess significant seeding activity and thus may be crucial for initiating the propagation of Aβ pathology during AD pathogenesis. In support of this theory, Aβ43 is common in diffuse plaques and is preferentially found in the core region of amyloid Aβ plaques, suggesting that it might deposit early during plaque formation [27, 96]. Furthermore, Aβ43 is the earliest-depositing Aβ species in the brains of an AD transgenic mouse model that expresses mutant APP [98]. Certain mutations in presenilin-1, including the AD-causing L435F and R278I variants, also cause increased production of Aβ43 at the expense of Aβ40 and Aβ42 [34, 40, 59, 62, 76, 87, 91]. This supports a model in which early Aβ43 aggregate seeds drive the downstream formation and propagation of Aβ aggregates containing other Aβ species, such as Aβ42 and Aβ40. While there are conflicting results about the cross-seeding of Aβ42 by Aβ43 aggregates in vitro [10, 14], our results argue that Aβ43 aggregates can act as a scaffold for the aggregation of Aβ42 in vivo since Aβ42 levels and deposition were greatly increased in *App*^NL−F^ mice inoculated with Aβ43. Consistent with this notion, expression of Aβ43 in Drosophila triggers aggregation of the normally soluble Aβ40 [7].

Our findings suggest that targeting Aβ43-containing seeds, potentially via immunotherapy or by reducing Aβ43 production, may be an effective means of halting the propagation of Aβ aggregates in the early stages of AD. Indeed, Aβ42 peptide immunization studies in AD patients have revealed that Aβ43-positive plaques can be cleared without a concomitant increase in vascular Aβ43 deposition [28], and the increased Aβ43 levels generated by mutant presenilin-1 alleles can be counteracted using small molecule γ-secretase modulators [89]. The therapeutic antibody aducanumab, which selectively recognizes Aβ aggregates including soluble oligomers and insoluble fibrils [81], is able to intercept early pre-amyloid Aβ seeds and reduce the development of cerebral Aβ pathology in mice [90], revealing that targeting Aβ seeds may have clinical benefit in AD patients.

## Supplementary Information

**Additional file 1**: **Supplementary Table 1.**

**Additional file 2**: **Supplementary Fig. 1.** Determining the presence of protease-resistant Aβ species in the brains of Aβ42- and Aβ43-inoculated *App*^NL−F^ mice. Immunoblot of insoluble, PK-resistant Aβ species in brain homogenates from *App*^NL−F^ mice inoculated with PK-resistant recombinant Aβ42 (**a**) or Aβ43 (**b**) aggregates. Aβ was detected using the antibody 82E1

**Additional file 3**: **Supplementary Fig. 2.** Spontaneous Aβ deposition in *App*^NL−F^ mice. **a** Schematic of the location of spontaneous Aβ pathology in the brains of *App*^NL−F^ mice at ~7.5 months of age. **b** Quantification of Aβ42 plaques (number of plaques per sagittal section) in the frontal/parietal cortex of inoculated *App*^NL−F^ mice at 6 months post-inoculation with either Aβ38 (n = 6), Aβ40 (n = 8), Aβ42 (n = 10), Aβ43 (n = 8), TgCRND8 Aβ (n = 9), or *App*^NL−F^ Aβ (n = 9). Mice inoculated with either dH_2_O (n = 5) or material derived from a non-Tg mouse brain (n = 6) were used as negative controls. There was no significant difference between the groups of inoculated mice (*P* = 0.69 by a Kruskal–Wallis test). Open circles indicate female animals and filled circles indicate male animals. **c** Representative images of small Aβ42 plaques (red arrows; 12F4 immunohistochemistry) in the frontal/parietal cortex of *App*^NL−F^ mice at 6 months post-inoculation with either dH_2_O or PK-resistant recombinant Aβ aggregates. Scale bar = 50 µm (applies to all images)

**Additional file 4**: **Supplementary Fig. 3.** Deposition of full-length Aβ species in the brains of Aβ-inoculated *App*^NL−F^ mice. Representative images of full-length Aβ deposition (82E1 immunohistochemistry) in the indicated brain regions of *App*^NL−F^ mice at 6 months post-inoculation with either PK-resistant recombinant Aβ43 aggregates, purified TgCRND8 Aβ aggregates, or purified *App*^NL−F^ Aβ aggregates. Scale bar = 50 µm (applies to all images)

## Data Availability

All data generated or analyzed during this study are included in this published article.
