# Supplementary material for: Aβ43 aggregates exhibit enhanced prion-like seeding activity in mice
Source: Acta Neuropathol Commun. 2021 May 10;9:83. doi: 10.1186/s40478-021-01187-6 (PMC8112054; doi:10.1186/s40478-021-01187-6)
Supplement: Supplementary file 1 — Additional file 1: Supplementary Table 1. [file 40478_2021_1187_MOESM1_ESM.docx]

**Supplementary Table 1** List of inoculated *App*^NL-F^ mice with intercurrent illness removed from the study

| **Inoculum** | **Sex** | **Days post-inoculation** | **Notes** |
| --- | --- | --- | --- |
| dH2O | Female | 106 | Found dead in cage (no symptoms apparent) |
|  | Female | 181 | Found dead in cage (no symptoms apparent) |
|  | Male | 92 | Found dead in cage (no symptoms apparent) |
| recAβ38 | Female | 49 | Found dead in cage (no symptoms apparent) |
|  | Female | 57 | Found dead in cage (no symptoms apparent) |
|  | Female | 183 | Found dead in cage (no symptoms apparent) |
| recAβ40 | Female | 70 | Found dead in cage (no symptoms apparent) |
|  | Female | 91 | Found dead in cage (no symptoms apparent) |
| recAβ43 | Female | 68 | Found dead in cage (no symptoms apparent) |
| Non-Tg | Female | 120 | Found dead in cage (no symptoms apparent) |
|  | Female | 146 | Found dead in cage (no symptoms apparent) |
|  | Female | 175 | Found dead in cage (no symptoms apparent) |
| TgCRND8 | Female | 119 | Found dead in cage (no symptoms apparent) |
