# Supplementary figures and images for: Aβ43 aggregates exhibit enhanced prion-like seeding activity in mice
Source: Acta Neuropathol Commun. 2021 May 10;9:83. doi: 10.1186/s40478-021-01187-6 (PMC8112054; doi:10.1186/s40478-021-01187-6)

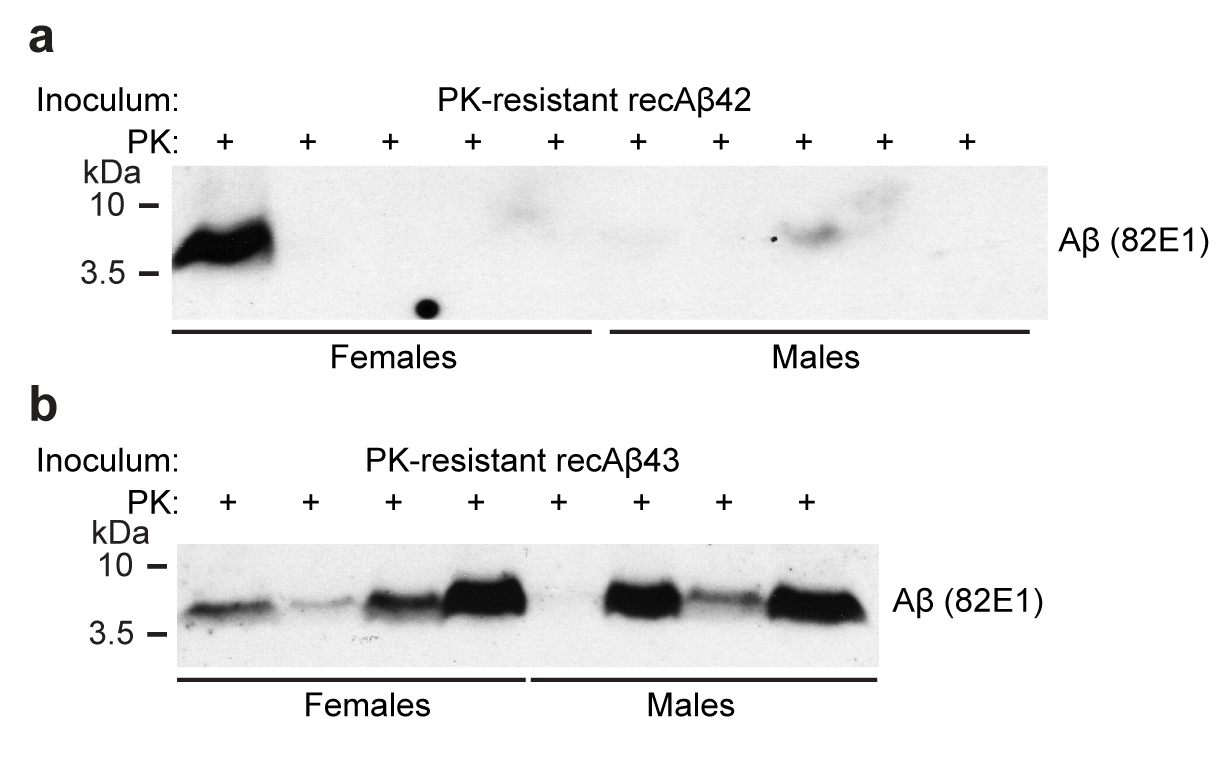

Supplement: Supplementary file 2 — Additional file 2: Supplementary Fig. 1. Determining the presence of protease-resistant Aβ species in the brains of Aβ42- and Aβ43-inoculated AppNL−F mice. Immunoblot of insoluble, PK-resistant Aβ species in brain homogenates from AppNL−F mice inoculated with PK-resistant recombinant Aβ42 (a) or Aβ43 (b) aggregates. Aβ was detected using the antibody 82E1 [file 40478_2021_1187_MOESM2_ESM.tif]

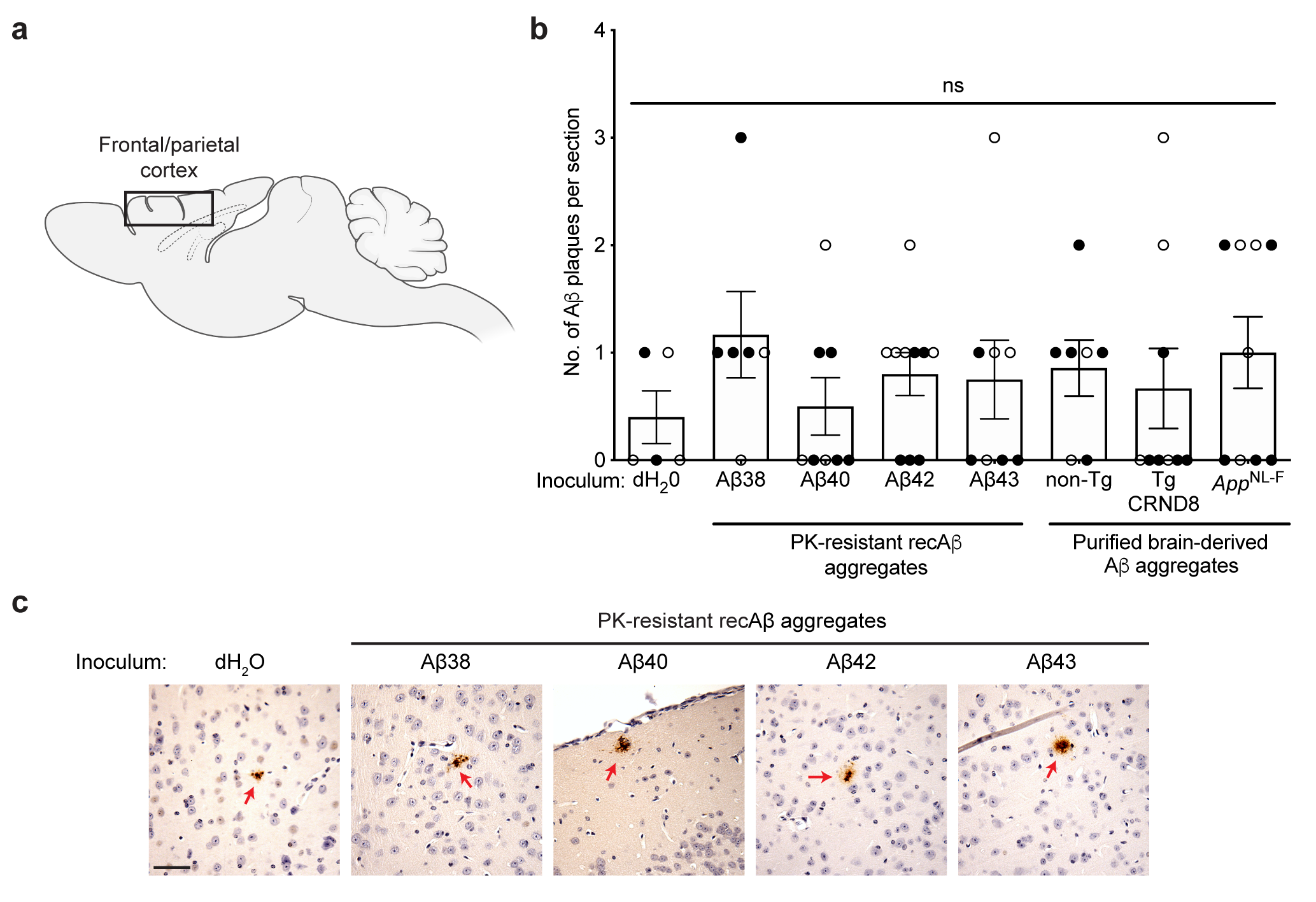

Supplement: Supplementary file 3 — Additional file 3: Supplementary Fig. 2. Spontaneous Aβ deposition in AppNL−F mice. a Schematic of the location of spontaneous Aβ pathology in the brains of AppNL−F mice at ~7.5 months of age. b Quantification of Aβ42 plaques (number of plaques per sagittal section) in the frontal/parietal cortex of inoculated AppNL−F mice at 6 months post-inoculation with either Aβ38 (n = 6), Aβ40 (n = 8), Aβ42 (n = 10), Aβ43 (n = 8), TgCRND8 Aβ (n = 9), or AppNL−F Aβ (n = 9). Mice inoculated with either dH2O (n = 5) or material derived from a non-Tg mouse brain (n = 6) were used as negative controls. There was no significant difference between the groups of inoculated mice (P = 0.69 by a Kruskal–Wallis test). Open circles indicate female animals and filled circles indicate male animals. c Representative images of small Aβ42 plaques (red arrows; 12F4 immunohistochemistry) in the frontal/parietal cortex of AppNL−F mice at 6 months post-inoculation with either dH2O or PK-resistant recombinant Aβ aggregates. Scale bar = 50 µm (applies to all images) [file 40478_2021_1187_MOESM3_ESM.tif]

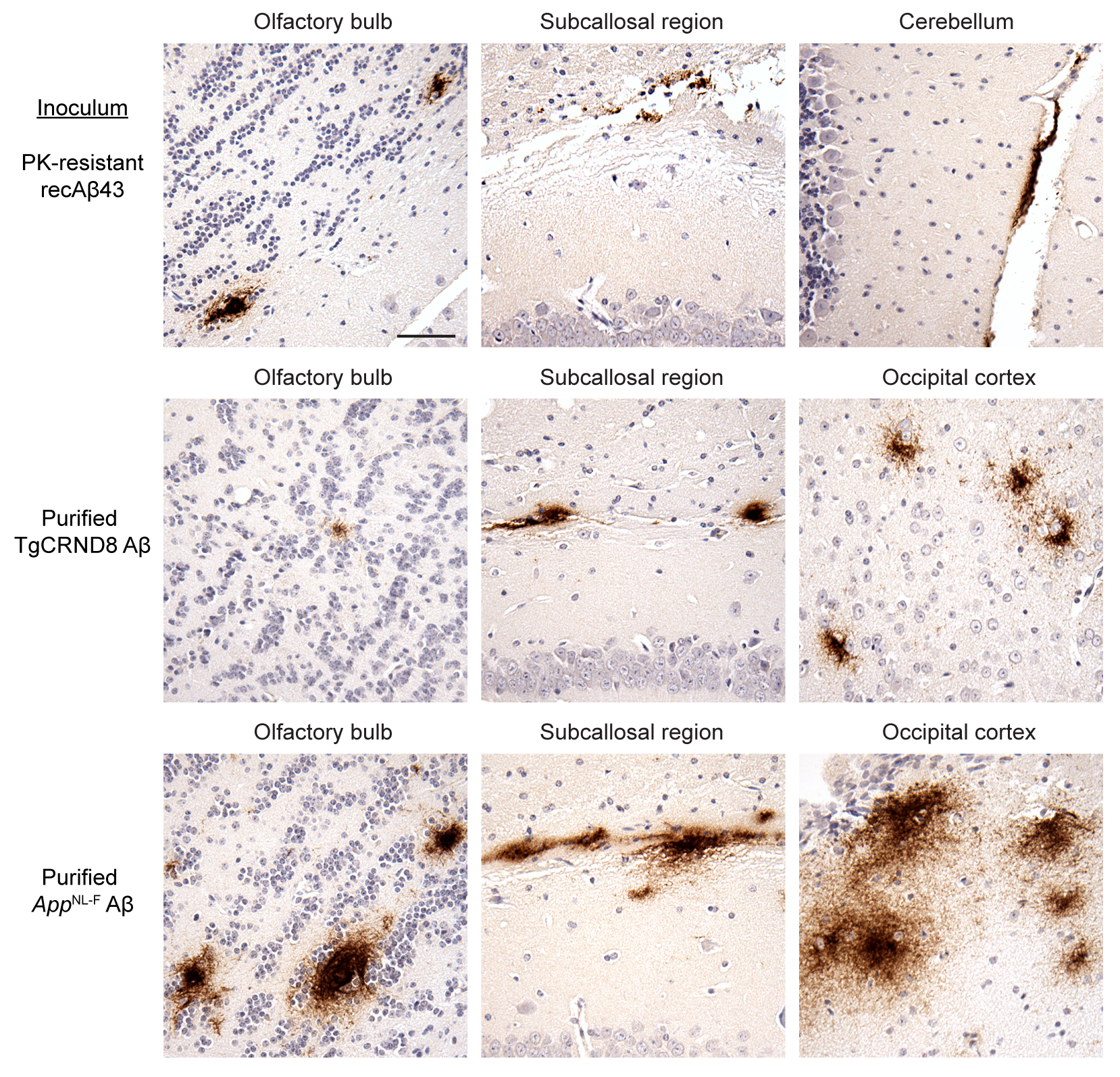

Supplement: Supplementary file 4 — Additional file 4: Supplementary Fig. 3. Deposition of full-length Aβ species in the brains of Aβ-inoculated AppNL−F mice. Representative images of full-length Aβ deposition (82E1 immunohistochemistry) in the indicated brain regions of AppNL−F mice at 6 months post-inoculation with either PK-resistant recombinant Aβ43 aggregates, purified TgCRND8 Aβ aggregates, or purified AppNL−F Aβ aggregates. Scale bar = 50 µm (applies to all images) [file 40478_2021_1187_MOESM4_ESM.tif]
